# The Impact of Item Difficulty on Judgment of Confidence—A Cross-Level Moderated Mediation Model

**DOI:** 10.3390/jintelligence11060113

**Published:** 2023-06-07

**Authors:** Yuke Zhou, Ning Jia

**Affiliations:** College of Education, Hebei Normal University, Shijiazhuang 050024, China

**Keywords:** judgment of confidence, item difficulty, processing fluency, intelligence, cross-level moderated mediation model

## Abstract

The factors that influence metacognitive judgments often appear in combination, rather than in isolation. The multi-cue utilization model proposes that individuals often make use of multiple cues when making judgments. Previous studies have focused on the integration of intrinsic and extrinsic cues, while the current investigation examines the integration and influence of intrinsic cues and mnemonic cues. Judgment of confidence is a common form of metacognitive judgment. In this study, 37 college students completed Raven’s Progressive Matrices and made judgments of confidence. We used the cross-level moderated mediation model to explore the impact of item difficulty on confidence judgments. Our results indicated that item difficulty negatively predicts the level of confidence. Item difficulty has an impact on the confidence evaluation by altering the processing fluency of intermediate variables. The joint effect of intrinsic cue item difficulty and mnemonic cue processing fluency influences confidence judgments. Additionally, we found that intelligence moderates the effect of difficulty on processing fluency across levels. Specifically, individuals with higher intelligence exhibited lower fluency on difficult tasks and higher fluency on simple tasks than individuals with lower intelligence. These findings expand on the multi-cue utilization model and integrate the influence mechanism of intrinsic and mnemonic cues on confidence judgments. Finally, we propose and verify a cross-level moderated mediation model that explains how item difficulty affects confidence judgments.

## 1. Introduction

Judgment of confidence (JOC) is a typical retrospective metacognitive monitoring, which involves assessing one’s own certainty in responses after completing a task (Schraw 2008). However, during this evaluation, individuals may display tendencies of overconfidence or a lack of confidence, leading to a disparity between perceived and actual performance and affecting the accuracy of individual metacognitive monitoring (Baranski and Petrusic 1994; Çapan et al. 2022; Jackson et al. 2017; Putnam et al. 2022). Various factors, such as experience (Cichoń et al. 2018), feedback (Iida et al. 2020), and effort (Lee and Daunizeau 2021) can affect individuals’ confidence judgments. The most important and common influencing factor is the item difficulty (Arnold et al. 2017; Clariana and Park 2021). The item difficulty significantly impacts the accuracy and confidence level of individual judgments, leading to a “hard-easy effect” on the accuracy of confidence judgments. Specifically, individuals tend to exhibit greater overconfidence in more challenging tasks, while the phenomenon of overconfidence tends to diminish or be replaced by a lack of confidence in relatively easier tasks (Griffin and Brenner 2004; Juslin et al. 2000; Lamotte et al. 2017). Although limited research has been conducted on the degree of JOC in decision making, Jeckeln et al. (2022) employed a confidence-based forced-choice paradigm to investigate facial recognition. Participants were presented with three faces, one of which was inconsistent with the others, and the task difficulty was manipulated by adjusting lighting angles. Following task completion, participants were required to indicate the experiment in which they had higher confidence. The findings revealed that task difficulty significantly impacted confidence levels, with higher ease resulting in higher levels of confidence. Specifically, the study demonstrated that lower item difficulty was associated with higher levels of confidence. The magnitude of JOC is an essential indicator of metacognitive monitoring (Boldt and Gilbert 2022). Research on confidence levels helps to obtain intuitive metacognitive results and separate them from achievements and accuracy, facilitating further exploration of the factors influencing the accuracy of metacognitive monitoring. Therefore, this study investigates the relationship between task difficulty and confidence judgment, and further explores how task difficulty affects metacognitive monitoring.

Koriat (1997) proposed a cue utilization model that suggests individuals influence judgment results by using different cues in tasks, including intrinsic cues, extrinsic cues, and mnemonic cues. Intrinsic cues refer to item characteristics that individuals perceive as a priori indicators of learning difficulty, such as font size. Extrinsic factors include task conditions or coding operations used by individuals, such as task frequency. Mnemonic cues are based on existing cue information of the item, such as processing fluency and availability of information. Both intrinsic and extrinsic cues can directly affect individual metacognitive judgments and may also play an indirect role by influencing mnemonic cues. Hertzog et al. (2013) extended Koriat’s theory by proposing a multi-cue utilization model that advocates for exploring the relative contribution of independent variables carrying different metacognitive cues to the judgments. They suggested considering the joint effect of different cues on the level of metacognitive judgment. Wang (2019) reviewed that people can integrate multiple extrinsic and intrinsic cues in metacognitive judgment. However, there are fewer studies on the integration of intrinsic and extrinsic cues with mnemonic cues. The impact of both extrinsic and intrinsic cues on the levels of metacognitive judgment via mnemonic cues has been confirmed. Prior research classified mnemonic cues as intrinsic cues to cognitive processing but did not consider their role in cue integration (Chen et al. 2016; Susser et al. 2013; Wang and Qu 2019). The purpose of this study is to integrate intrinsic cues and mnemonic cues into the analysis and explore the underlying mechanisms of cognitive processes through the integration of cues. This will help to further consider the possible existence of responses in cognitive processes from the perspective of cue utilization.

Processing fluency is a more frequently utilized mnemonic cue (Reber and Greifeneder 2016; Rhodes and Castel 2008). It refers to how easily individuals can process presented information (Alter and Oppenheimer 2009). According to the fluency hypothesis, processing ease can unconsciously influence individual judgments (Undorf et al. 2017). Processing time, which is the time it takes for an individual to complete a task (Dodonova and Dodonov 2013), is often used as a measure of processing fluency (Bahnik 2019; Reber et al. 2004; Winkielman et al. 2015). Generally, longer processing times indicate lower processing fluency. Previous research has often focused on single factors while overlooking the relationship and interaction between mnemonic cues and intrinsic perceptual cues. Rhodes and Castel (2008) conducted a study using various font sizes to examine their impact on people’s judgments. They focused on the influence of perceptual cues on metacognitive judgments, while ignoring the related mnemonic cues. Additionally, there are investigations that analyze these two aspects as separate factors. Voodla and Uusberg (2021) investigated the effects of item difficulty and fluency on confidence judgments. Their study found that confidence levels decrease with increasing task difficulty. Confidence judgments were significantly lower for difficult tasks than for easy tasks. On easy tasks, high fluency items led to significantly higher confidence judgments than low fluency items. However, there was no significant difference in confidence judgments between high and low fluency levels for difficult items. The experiment focused on how two factors affect metacognitive judgment and ignored how item difficulty affects processing fluency. The relationship between item difficulty and processing fluency has been proposed by Shulman et al. (2022), who suggest that language complexity affects processing fluency. Complex language leads to a less fluent experience, and individuals have higher levels of fluency when processing easier words. Additionally, answering more difficult questions generally requires a longer processing time (Liao 2018; Yang et al. 2002). De Martino et al. (2013) have suggested that people’s confidence in decision making is influenced by the duration of response time. Longer response times tend to result in lower confidence levels for individuals. In other words, higher fluency levels tend to be associated with higher confidence levels. To sum up, individuals may perceive processing as easy or difficult due to intrinsic cues during the task. For difficult questions, individuals may feel that the task is difficult and have a low degree of fluency. However, when information is easy to process, a smooth perceptual experience is generated, and the degree of fluency is high. This sense of fluency can serve as a cue for judgment (Bahnik 2019), which in turn affects JOCs. Our hypothesis (H1) suggests that the combination of intrinsic and mnemonic cues influences metacognitive judgment, with processing fluency acting as a mediating factor between item difficulty and JOC magnitude.

Based on decision making theory, both individual and item level factors influence the amount of time a person takes to process a problem (Lindquist 2019). As a result, the level of processing fluency is influenced not only by cues at the item level but also varies among individuals. Research has shown that people with higher intelligence tend to have shorter processing times, indicating higher fluency (Jensen 2006). Brewer and Unsworth (2012) found that individuals with lower cognitive ability may experience more retrieval failures or put forth less effort when faced with difficult items. Moreover, general intelligence factors have been found to impact decision making speed and interact with factors that affect decision making difficulty. Item difficulty moderates the correlation between individual general intelligence and reaction time (Willoughby and Lee 2021). These finding results indicated that the processing time during individual decision making is influenced by the interaction between intelligence and task difficulty. Furthermore, the correlation between general cognitive ability and processing time demonstrates an increasing trend as task complexity grows (Schmitz and Wilhelm 2016). In conclusion, intelligence moderates the impact of the item on the task. We attempted to combine variables at both the item and individual levels to investigate their impact on cognitive tasks. While exploring the influence of intrinsic cues, we considered the role of individual differences in cognitive processes. To reduce interference among variables, individual factors were separated as a second-level variable. Furthermore, we speculated that, during the task, the difficulty of the task would affect an individual’s fluency and be moderated by their intelligence. Therefore, our hypothesis (H2) suggests that individual-level intelligence plays a moderating role in the effect of item difficulty on processing fluency.

**Figure 1:**
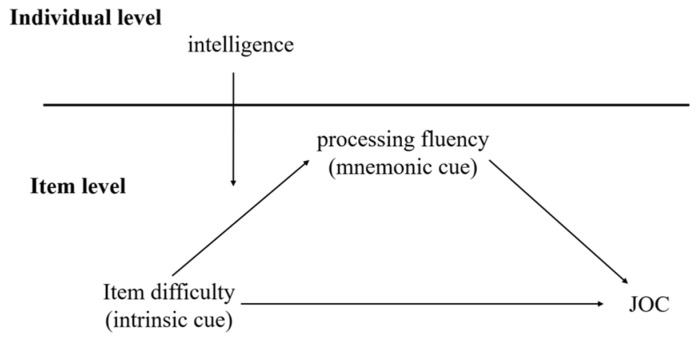
A cross-level moderated mediation model in which item difficulty affects judgment of confidence (JOC) magnitude.

In summary, this study proposes a moderated mediation model grounded in decision theory, as illustrated in Figure 1. We investigate the mediating effect of item-level processing fluency and the moderating effect of individual-level intelligence factors, with the aim of revealing the impact of integrating multiple cues on metacognitive judgments.

## 2. Materials and Methods

### 2.1. Participants

In this research, 40 adults (20 males and 20 females, age range 20–26, M_age_ = 22.83, SD_age_ = 1.38) were recruited from a college. The participants in this study had normal or corrected vision, basic computer skills, and had not undergone any intelligence testing in the past two months. The participants were paid after completing the experiment. Three participants were excluded from the analysis due to their valid data not meeting the experimental criteria, resulting in 37 valid participants. The selection criteria in this study were to exclude participant individuals who had more than 15% outliers during the experimental process. Outliers included cases where participants failed to answer questions within the designated time frame or cases where they provided confident judgments despite not providing any response. The final retention rate was 89.1%.

### 2.2. Materials

The experimental materials used in this study included Raven’s Standard Progressive Matrices (SPM) and Raven’s Advanced Progressive Matrices (APM, Raven and Court 1938).

The SPM consists of 5 sections, each containing 12 visually presented geometric problems. The questions range from simple filling of missing images to abstract reasoning problems that increase in difficulty. The participants are required to select the correct missing item from 6 alternative answers in a 2 × 3 matrix or 8 alternative answers in a 2 × 4 matrix (Ismat and Sidiqui 2015). The difficulty level of each set of questions increases with the increase in the number of questions. The difficulty level also increases across different sets, and the order of difficulty is consistent with the original questions (Zhang and Wang 1989). The APM consists of 3 sets of 36 questions, each containing 12 questions. For this experiment, Group 4 and 5 of the SPM and Group 2 of the APM were selected as formal test questions, totaling 36 questions. Additionally, the first 6 questions in Group 3 of the SPM were chosen as practice questions.

### 2.3. Task Procedure

The experiment was comprised of two stages: a practice stage and a formal experiment stage. Each participant completed two tasks for each question, namely the question-answering task and the JOC task. The answering task was presented first, with the questions of each group presented sequentially on the screen. Each question was displayed for one minute, and participants were required to answer them within that time frame by pressing the corresponding number keys on the keyboard (1–8 corresponding to answer options). Immediately after answering each question, participants proceeded to the JOC task, where they were asked to evaluate their confidence levels in providing a response to the inquiry within a time limit of 3 s. The confidence judgment was comprised six levels: 0 = 0%, 1 = 20%, 2 = 40%, 3 = 60%, 4 = 80%, and 5 = 100%, with each numerical value corresponding to the probability that the participant believed they were correct. Participants made their judgments by pressing the corresponding number keys. After completing the confidence judgment for a question, participants moved on to the answering stage of the next question and repeated the process until all 36 questions were answered.

### 2.4. Statistical Analyses

We used HLM 6.08 to construct multiple regression paths and the final cross-layer mediation model. According to previous research, in multilevel data analysis, it is appropriate to conduct hierarchical linear regression modeling (HLM) when the number of level 1 samples is greater than 30 and each level 1 sample corresponds to a level 2 sample size greater than 30 (Kreft 1996; McNeish and Stapleton 2014). In this study, the number of individual-level participants was 37, and the number of item-level samples was 36, which meets the criteria for analysis. Additionally, we utilized IBM SPSS 24.0 to obtain the relative accuracy of confidence judgments and further verify the mediation effect.

## 3. Results

### 3.1. Calculating Variables

Intelligence: The indicator of intelligence is the score on the SPM. One point was awarded for each correct answer, and no points were awarded for incorrect answers. The sum of all scores for the questions represented an individual’s intellectual score.

Item difficulty: Item difficulty coefficient was used as a measurement indicator for difficulty. The standard practice of measuring difficulty (*p*-value) was followed in this study, where the correct pass rate of all subjects was used as the difficulty coefficient.

Processing fluency: Processing time was used as a measurement indicator for processing fluency. The longer the processing time, the worse the fluency.

JOC relative accuracy: The relative accuracy of JOC can be determined using Gamma-related laws as proposed by Nelson (1984). The JOC’s relative accuracy pertains to the predictive ability of one item compared to another, with the Gamma value representing the G correlation coefficient between confidence judgments and test scores. The Gamma value ranges from −1 to 1, with higher values indicating greater accuracy. A single sample T-test revealed that the average Gamma value for JOC was 0.733, with T (34) = 21.178 and *p* < 0.001, and Cohen’s d = 3.58. These results indicate that the trial JOC significantly overestimates accuracy.

JOC magnitude: The indicator was used to measure the confidence level of the participants, which was composed of six levels: 0–5.

### 3.2. Descriptive Statistics and Correlational Analysis Results

The means, standard deviations, and correlation coefficients of each variable are presented in Table 1. We conducted a correlation analysis between the item difficulty, the processing fluency, and the level of confidence in solving the problem. Specifically, there existed a significant negative correlation (r = −0.624, *p* < 0.01) between item difficulty and processing fluency, while a significant positive correlation (r = 0.383, *p* < 0.01) existed between item difficulty and JOC magnitude. Furthermore, a significant negative correlation (r = −0.364, *p* < 0.01) was observed between processing fluency and JOC magnitude.

### 3.3. Mediation Effect Analysis

A total of 37 participants completed 36 test questions, and data from the item containing missing values were deleted. Finally, 1283 data points were retained to form an individual, with a two-level nested relationship of the items. Hierarchical linear model (HLM) was used for data analysis, where the first level represents an item variable, the second level represents an individual variable, and the control variables include individual gender and age. First, JOC magnitude was used to establish zero models for variables and test zero model inspection. The results showed that the differences in the group (σ^2^) and the meter (т00) were 1.195 and 0.657, respectively. The calculated intra-class correlation coefficient (ICC) for JOC was 35.5%, indicating significant between-group differences (F (36, 1247) = 4.24, *p* < 0.001). Furthermore, the estimated reliability ICC (2) was 0.950, which exceeded the recommended threshold of 0.7. Based on these conditions, a multi-layered linear analysis could be conducted.

The proposed hypothesis(H1) posits that processing fluency acts as a mediator in the relationship between item difficulty and JOC magnitude. To investigate this, a hierarchical linear model was constructed to examine the primary effect of item difficulty on JOC magnitude at the item level. The step-up regression method was then used to test for the mediating effect. The first step involved testing the main effects of the predictor variables. Results from Model 2 indicated that item difficulty (γ = 2.19, p < 0.001) significantly predicted JOC magnitude, while controlling for the effects of age and sex at the individual level. Consequently, condition 1 of the mediation effect was established.

We examined the impact of the independent variable, item difficulty, on the intermediate variable, processing fluency. Taking processing fluency as the dependent variable, the results of zero model 2 of HLM showed that the intra-group variance (σ^2^) and the inter-group variance (т00) were 144.35 and 13.06. The intra-group correlation coefficient ICC (1) calculated for processing fluency was 8.3%, and the inter-group variance was significant, F (36, 1247) = 18.18, *p* < 0.001. The reliability estimate ICC (2) was 0.758 > 0.7, which indicates the suitability of the data for multilayer linear analysis. Controlling for individual age and sex, item difficulty (γ = −36.15, *p* < 0.001) significantly predicted processing fluency.

In the regression equation where both the independent variable, item difficulty, and the mediating variable, processing fluency, were entered simultaneously, Model 5 results showed that processing fluency (γ = −0.03, *p* < 0.001) significantly predicted JOC magnitude. Compared to Model 2, the coefficient of item difficulty (γ = 1.23, *p* < 0.001) became smaller. Therefore, it was demonstrated that processing fluency played a significant mediating role in the relationship between item difficulty and JOC magnitude, and Hypothesis 1 was supported.

To demonstrate the existence of a mediating effect and ensure the consistency of the analysis results, this study continued to use the bootstrap method for verification. The analysis results showed that the mediating effect of processing fluency between item difficulty and JOC was 0.982, accounting for 43.8% of the total effect, and the 95% confidence interval was [0.6720, 1.3062]. A confidence interval that does not contain zero indicated the presence of a significant partial mediation effect. Therefore, these findings offer additional support for Hypothesis 1.

### 3.4. Cross-Level Moderating Effect Inspection

**Figure 2:**
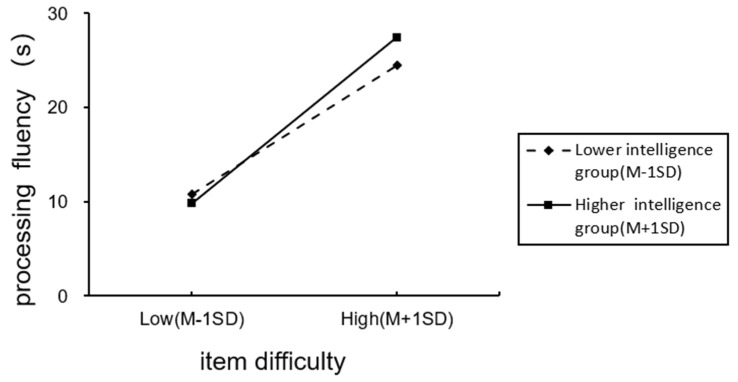
Moderating effect diagram.

To test the moderating effect of individual intelligence, we centralized the cross-level moderating variables of individual intelligence. Then we included controlled variables, the item difficulty, individual intelligence, and interactive items between individual intelligence and the item difficulty into the model. In Table 2, M7 showed that the cross-level interaction of individual intelligence had a significant negative effect on the processing fluency (γ = −1.26, *p* < 0.001). This result suggests that individual intelligence played a negative regulatory role in the relationship between difficulty and processing fluency. To provide a comprehensive explanation of the moderating effect, we added and subtracted one standard deviation from the mean value. This method enabled us to highlight the difference in the impact of item difficulty on processing fluency, based on varying levels of intelligence. To enhance clarity, we used “P” as a numerical indicator of the item difficulty, with larger values indicating harder items. Figure 2 shows the moderating effect diagram. Compared to individuals with low intelligence, those with high intelligence had a stronger positive effect on processing fluency in the context of harder items, supporting Hypothesis 2.

To further confirm the moderated mediating effect, PROCESS was used to analyze whether there was a difference in the mediating effect under the influence of different intelligence levels. The results are shown in Table 3. When individual intelligence was higher, processing fluency had a significant mediating effect between the item difficulty and JOC magnitude. The indirect effect is −1.106, with a 95% confidence interval of [−1.461, −0.756]. When individual intelligence was lower, processing fluency also had a significant mediating effect, and the indirect effect is −0.858, with a 95% confidence interval of [−1.165, −0.571]. Moreover, the difference between the two was significant, and the coefficient was 0.248. The 95% confidence interval is [0.298, 0.185]. These findings suggested that when the individual intelligence level was different, the mediating effect of the item difficulty on processing fluency and JOC magnitude differed. This result supported the general research hypothesis that individual intelligence, as a moderating variable, moderated the mediating effect of processing fluency.

## 4. Discussion

### 4.1. The Effect of Item Difficulty on JOC Magnitude

This study investigated the relationship between item difficulty and JOC magnitude, revealing the role of the item difficulty as an intrinsic cue that affects the level of confidence in an individual’s judgments. Specifically, we explored the relationship between item difficulty and metacognitive judgments, finding a negative correlation between item difficulty and JOC magnitude, which supports the notion that harder tasks can reduce individual confidence (Arnold et al. 2017; Clariana and Park 2021). Moreover, research suggests that the difficulty of objective items does indeed affect individuals’ subjective confidence, which is consistent with previous findings (Jeckeln et al. 2022). Unlike the accuracy of metacognitive judgments, easy items often elicit higher confidence levels than hard items at the item level, regardless of whether the question is answered correctly, which is a clear trend.

### 4.2. The Mediating Effects of Processing Fluency

Based on the cue utilization theory and the multi-cue utilization model, we proposed that mnemonic cue processing fluency plays a mediating role in the influence of intrinsic cues on metacognitive judgments. Our results showed that task item difficulty predicts the level of JOC directly or indirectly through the mediating role of processing fluency. Processing fluency plays a partially mediating role between item difficulty and confidence level. The hypothesis has been confirmed that under the joint action of multiple cues, the integration between intrinsic cues and mnemonic cues does have an impact on the results. Processing fluency cannot simply be combined with intrinsic cues for exploration. In our model, the mediating effect of processing fluency accounts for 43.8%, explaining the influence of item difficulty on confidence judgment to a large extent. This outcome is a crucial processing process that cannot be ignored.

Specifically, item difficulty negatively affects processing fluency, which in turn positively predicts JOC magnitude. Specifically, as the task difficulty increases, processing fluency decreases, leading to lower levels of confidence judgments. These findings indicate that intrinsic cues not only directly affect metacognitive judgments but also indirectly affect it through affecting mnemonic cues. Parts of the results are consistent with the study of De Martino et al. (2013). The current study highlights the importance of exploring the mechanisms of intrinsic cues on metacognitive judgment from the perspective of cue utilization. Additionally, these findings encourage researchers to treat mnemonic cues as separate cues to help identify stages in the processing process. Future studies can consider the influence of multi-cue integration between intrinsic, extrinsic, and mnemonic cues on the metacognitive judgment magnitude.

### 4.3. Cross-Level Moderating Effect of Intelligence

Processing fluency can be affected by task difficulty and individual differences. Although item difficulty can predict individual confidence directly or indirectly, the effect may vary depending on individual intelligence levels. Individuals with lower intelligence may be less affected by task difficulty. The study found that, compared to those with high intelligence, the mediating effect of processing fluency was lower in individuals with low intelligence, and the positive effect between difficulty and processing fluency was less apparent. This result suggests that intelligence, as an individual trait, can to some extent influence the relationship between item characteristics and task processes. Parts of the results of this study are consistent with the accuracy–intelligence–processing time study of Dodonova and Dodonov (2013), which found that the fluency and difficulty-related change rates expressed by individuals with high intelligence and those with low intelligence differed. The difference is that in this study, individual variables were separated from item variables, considering the impact of individual traits on task completion, thereby reducing the error caused by individual differences and making the results more accurate.

As shown in Figure 2, when the items are simple, there is little difference in fluency between individuals with high and low intelligence. These easy questions provide individuals with a high level of fluency, which leads to a higher magnitude of JOC. However, as the difficulty of the items increases, the fluency of individuals with higher intelligence had a greater impact, and the difference between the fluency of individuals with higher and lower intelligence increased. One possible explanation is that individuals with lower intelligence may choose to provide quick answers or not put in more effort when they perceive that the item difficulty is beyond their ability (Brewer and Unsworth 2012), resulting in poor performance. However, due to their short processing time and high fluency, they may be more confident in their answers. On the other hand, people with higher intelligence tend to work on solving difficult problems, resulting in less fluency and lower confidence levels, but with correct answers. Overall, the results show that students with lower intelligence tend to have lower inner standards, higher self-belief, and are less affected by fluency and process. Highly intelligent students, on the other hand, tend to put in more effort and think carefully about their answers, which affects their fluency and confidence levels.

The moderating effect of intelligence implies that the influence of various tasks on individuals partly depends on their individual characteristics. However, intelligence is a stable trait that is not easily changed. If we want to maximize task efficiency, we can start by changing the difficulty level of the task. For example, we can set different difficulty levels for different individuals based on their intelligence characteristics, which can increase their fluency experience and boost their confidence levels. Based on current research findings, we can actively consider the effects of individual differences while exploring task characteristics, which can lead to more comprehensive and enriched results. In summary, intelligence plays a crucial role in moderating the impact of item difficulty on processing fluency.

### 4.4. Research Innovation and Deficiency

This study delves into the intrinsic mechanism that underlies the relationship between intrinsic cues and JOC magnitude. It also examines the role of individual traits in completing items. The multi-cue utilization model is expanded to encompass intrinsic and mnemonic cues, and their respective proportions in the final metacognitive judgments are explored. Furthermore, the study investigates the impact of individual differences in intelligence on the thinking process. In addition, this study separates the individual level from the item response level, conducting cross-level analysis of data to explore the influence of individual-level factors on item-level.

However, it is important to acknowledge several limitations of this study that warrant further investigation in future research. Firstly, college students were specifically chosen as participants in this study to investigate the role of individual intelligence in cognitive tasks. Considering the stage-based and individual differences in intelligence development, it is crucial to investigate the role of intelligence across different age groups. For instance, the primary and secondary school stage is a critical period in human intelligence development, characterized by significant individual differences (Breit et al. 2020). Therefore, it is highly necessary to further examine the influence of intellectual characteristics of primary and secondary school students on various tasks. Future research could also explore the moderating effect of intelligence levels at different developmental stages. Secondly, the SPM test was utilized as an intelligence measure in this study, primarily emphasizing individuals’ visual reasoning ability. To obtain a comprehensive understanding of intelligence, future research should incorporate different types of intelligence tests to explore the effects of other intelligence components. Thirdly, processing fluency was utilized as a mnemonic cue to explore the potential association between intrinsic cues and metacognitive judgment in this study. In future research, investigators could examine how the integration of different categories of cues, such as information availability, influences metacognitive judgment. Finally, intelligence was utilized as a moderating variable in the study. In the future, other individual-level variables, such as cognitive style and motivation, could be considered for further investigation.

## 5. Conclusions

This study provides evidence for the integration of cues from the perspective of cue utilization theory and demonstrates the role of mnemonic cues throughout the process. Individuals use both intrinsic cues and mnemonic cues when making confidence judgments. The current research results also highlight the impact of intelligence on completing cognitive tasks, and we should pay special attention to the important role of multiple cue integration and individual differences in the cognitive process. In conclusion, item difficulty affects the confidence level through the intermediate variable processing fluency, and intelligence is the primary cross-level moderating variable, so a cross-level moderating mediation model is constructed.

## Figures and Tables

**Table 1:** The mean, standard deviation, and correlation coefficient of each variable.

Variable	M	SD	1	2	3
Individual level					
Age	22.76	1.38			
Gender	1.46	0.51			
Intelligence	26.32	3.58			
item level					
Item difficulty	0.74	0.22	—		
Processing fluency	18.14	12.54	−0.624 **	—	
JOC magnitude	4.24	1.35	0.383 **	−0.364 **	—

Note: ** *p* < 0.01, 1 = female, 2 = male; intelligence is level 2 data, so there is no correlation coefficient with level 1 data.

**Table 2:** Assumption analysis.

Variable	Processing Fluency				JOC Magnitude		
	M3 (Null)	M4	M6	M7	M1 (Null)	M2	M5
Intercept (γ_00_)	18.18 ***	35.68 ***	8.22	10.13	4.24 ***	3.53 *	4.89 **
Gender		−0.50	−0.47	−0.45		−0.52	−0.58 *
Age		0.44	0.38	0.38		0.01	0.01
Item difficulty		−36.15 ***	−2.48 ***	−36.12 ***		2.19 ***	1.23 **
Processing fluency							−0.03 ***
Intelligence			−1.28 *	−0.14			
Intelligence × item difficulty				−1.26 *			
σ^2^	144.35	76.60	76.57	76.57	1.20	0.83	0.73
m_00_	13.07 ***	106.63 ***	95.69 ***	14.60 ***	0.66 ***	3.32 ***	4.28 ***

Note: * *p* < 0.05, ** *p* < 0.01, *** *p* < 0.001. σ^2^ represents the within-group variance, and m_00_ represents the between-group variance. Non-standardized regression coefficients are the regression coefficients expressed in the unit of robust standard deviation.

**Table 3:** The mediating role of the moderating effect.

Intermediary Variables	Intelligence	Coefficient	95% Confidence Range	
			Lower Limit	Upper Limit
Processing fluency	High	−1.106	−1.461	−0.756
	Low	−0.858	−1.165	−0.571
	Difference	0.248	0.298	0.185

## Data Availability

The data presented in this study are available from the corresponding author upon request at jianing@hebtu.edu.cn. The data is not shown due to participant privacy.
